# Parental Involvement in Sport: Mother- and Father-Initiated Motivational Climates and Their Associations with Grit in Youth Male Team Sport Players

**DOI:** 10.3390/sports13120421

**Published:** 2025-12-01

**Authors:** Patrícia Coutinho, Cristiana Bessa, Isabel Mesquita, António M. Fonseca

**Affiliations:** CIFI2D, Centre of Research, Education, Innovation and Intervention in Sport, Faculty of Sport, University of Porto, Rua Dr. Plácido Costa, 91, 4200-450 Porto, Portugal; cbessa@fade.up.pt (C.B.); imesquita@fade.up.pt (I.M.); afonseca@fade.up.pt (A.M.F.)

**Keywords:** parental motivational climate, grit, consistency of interests, perseverance, team sports, youth sport

## Abstract

This study examined the relationship between perceived mother- and father-initiated motivational climate and grit in youth male team sports players. A total of 336 players from five team sports (basketball, football, handball, volleyball, and water polo) completed the Parent-Initiated Motivational Climate Questionnaire-2 and the Short Grit Scale. Multiple linear regressions showed that parental motivational climates explained between 8% and 16% of the variance in grit dimensions (R^2^ = 0.08–0.16, *p* < 0.001). Specifically, ego-oriented climates (worry-conducive and success-without-effort) was positively associated with consistency of interests (β = 0.20–0.27, *p* < 0.01), whereas mastery-oriented climates (learning/enjoyment) were negatively associated with this dimension (β = −0.09 to −0.26, *p* < 0.05). Conversely, both learning/enjoyment- and worry-conducive climates were positively associated with perseverance of effort (β = 0.26–0.37, *p* < 0.001), while success-without-effort climates had negative associations (β = −0.16, *p* < 0.01). When analyses were performed by sport, significant models were found for basketball, football, and volleyball (R^2^ = 0.07–0.34, *p* < 0.05), but not for handball or water polo. These findings underscore the differentiated and context-dependent associations of mothers’ and fathers’ motivational climates on the two dimensions of grit, providing evidence-based insights for parents, coaches, and sport organizations aiming to promote perseverance and long-term engagement in youth sport. By fostering appropriate motivational climates, stakeholder efforts towards more adaptive motivational climates may be tentatively associated with higher grit indicators, thereby contributing to developmental and psychological outcomes relevant to sustained participation in sport.

## 1. Introduction

Originally conceptualised by Duckworth and colleagues as the sustained perseverance and enduring commitment to long-term goals [1], grit refers to an individual’s capacity to persist with effort and maintain interest over extended periods despite obstacles or setbacks [1,2,3]. It comprises perseverance of effort (persistence and resilience in goal pursuit) and consistency of interests (the maintenance of focus and commitment over time), and has increasingly been examined as a relevant individual difference characteristic within sport psychology [4,5,6,7]. Empirical evidence has consistently associated grit with adaptive achievement behaviours and positive performance outcomes across multiple life domains, including sport [8,9,10,11,12]. Within athletic contexts, grit has been linked to greater investment in training and competitive activities [13,14], more deliberate practice [12,15,16], enhanced decision-making proficiency [8,17], higher performance [8,17] and stronger dedication to one’s sport [14,18]. Conversely, athletes with higher levels of grit are less likely to consider quitting their sport or switching to other activities [6,14,18], and research has also shown associations with additional adaptive psychological indicators such as athletic identity [19], self-restraint, and impulse control [15]. Given the breadth of evidence connecting grit to performance-relevant behaviours in sport, current research has begun to shift towards identifying antecedents associated with grit development. One proposed pathway emphasises the role of social-contextual influences, whereby messages and experiences conveyed by significant others, particularly parents, coaches and peers, may be associated with the extent to which athletes pursue long-term goals with perseverance and passion, with the motivational environment they create emerging as a potentially critical determinant [9,20,21].

Based on the assumptions of Achievement Goal Theory [22], motivational climates are created through socialising agents’ perspectives, behaviours and communications, which are theorised to influence individuals’ cognitive processes and behaviours within achievement contexts [9,21,23,24]. These climates are commonly described as mastery-oriented (task-involving) or ego-oriented (performance-involving). A mastery-oriented climate is characterized by the involvement of significant others (e.g., parents, coaches) who define success in terms of self-improvement, learning, and sustained effort [25,26]. Such environments typically promote adaptive outcomes, including persistence, self-esteem, intrinsic motivation, and performance [9,26,27]. Conversely, an ego-oriented climate fosters social comparison as the basis for success evaluation, often being associated with controlled motivation, anxiety, perfectionism, and dropout [26,28,29].

Complementary to AGT, the Self-Determination Theory (SDT) [30,31] offers a broader understanding of how these motivational climates may be associated with the quality of athletes’ motivation. SDT posits that the satisfaction of three basic psychological needs: autonomy (acting with volition and psychological freedom), competence (feeling capable and effective), and relatedness (feeling connected and valued by others), which are essential for sustaining optimal motivation and engagement. When these needs are fulfilled, individuals experience autonomous motivation, engaging in activities for their inherent satisfaction and personal relevance; when thwarted, motivation becomes controlled, driven by external pressures or contingencies. In sport settings, autonomy-supportive and mastery-oriented climates have been associated with greater basic psychological need satisfaction, which in turn has been linked with enjoyment, persistence, and adaptive functioning, whereas controlling or ego-involving climates have been associated with higher pressure, anxiety, and potential withdrawal [20,32,33].

Integrating AGT and SDT perspectives provides a coherent framework for understanding how parent-initiated motivational climates may be associated with the development of grit in young athletes. Despite the growing recognition of this connection, limited research has directly examined the associative role of parental motivational climates in relation to grit. To the best of our knowledge, only two studies have been conducted examining the association between grit and the motivational climate provided by teachers [34] and coaches [9]. Akin and Arslan [34] investigated the associations between the achievement goal orientations presented by university teachers and the predisposition towards grit in a sample of male and female undergraduate students. They found that task goal orientation was positively associated with students’ levels of grit. Despite the study’s significance, it was conducted within a non-sport context, focusing on non-sport agents (teachers and students), thus lacking specific insights into the relationship between motivational climates and grit in sports. More recently, Albert and colleagues [9] examined the relationships of the perceived motivational climate created by coaches (task- and ego-involving) and athletes’ goal orientation (task vs. ego) to grit predispositions among male adolescent soccer players. They found that a task orientation, but not the perception of a task-involving climate, was positively associated with higher levels of grit. Also, ego-related constructs are not significantly related to grit. While the findings of this study significantly contribute to advancements in this field, its focus is limited to analysing athletes’ perceptions solely concerning the motivational climate created by the coach. It lacks insights into perceptions about the motivational climate established by other influential social agents, such as parents.

Consolidated research has demonstrated that the parent-initiated motivational climate, referring to the socioemotional environment created by parents, significantly influences athletes’ motivation, perceptions, and behaviours [20,35,36,37]. Studies have consistently shown that a mastery-oriented climate is positively and significantly associated with athletes’ higher levels of intrinsic motivation, task enjoyment, persistence, and adaptive achievement behaviours [20,38]. On the other hand, an ego-oriented climate is often associated with high levels of extrinsic motivation [20,24], increased anxiety [39], perfectionistic cognitions [29] and burnout [29,40].

To the best of our knowledge, however, no studies to date have explored the relationships between the motivational climate established by parents and athletes’ predispositions towards grit. Considering that parents are considered key social agents in a child’s psychological and emotional development, the motivational climate fostered at home may be associated with differences in athletes’ determination, resilience and ability to sustain effort over time, all of which are elements of grit. Previous research examining related constructs, such as resilience and mental toughness, has already shown that motivational climates created by significant others (including parents) have been associated with these traits in youth athletes. For example, studies have found that supportive, mastery-oriented environments are positively associated with athletes’ levels of resilience and mental toughness, both of which share conceptual overlap with grit, particularly in the domains of sustained effort and coping with adversity [20,24,35]. Thus, investigating the correlation between parental motivational climates and an athlete’s grit can contribute to a more comprehensive understanding of how grit is developed and sustained in sporting contexts. This research is particularly significant in youth sports, especially concerning adolescent athletes, as this stage is critical for shaping identity, values, and beliefs, including those associated with sports participation [41]. Understanding the associations between parental motivational climate and grit during this specific phase is of pivotal importance.

Additionally, the understanding that grit predispositions vary across sports and cultural contexts [4,12,14] emphasizes the importance of exploring specific sporting contexts, particularly team sports. Often categorized as late specialization sports [42], team sports possess unique developmental features during adolescence, leading to a spectrum of psychological and emotional experiences and outcomes [43,44]. In this context, parents are considered relevant social agents whose behaviours may be associated with more or less adaptive experiences in sport, which may in turn relate to athletes’ grit predispositions. While studies have examined the motivational climate initiated separately by both fathers and mothers [45,46,47], the collective evidence fails to provide a nuanced understanding of the associations of each parent with athletes’ predispositions towards grit. This knowledge is critical in offering evidence-based guidelines and recommendations for parents, coaches, and organizations to establish more supportive and growth-oriented sporting environments.

### Objectives and Hypothesis

This study is a follow-up to a previously published investigation that examined the relationship between parent-initiated motivational climates and fear of failure in youth male team sport athletes [43]. While both studies draw upon the same dataset, they address distinct research questions and explore separate psychological constructs. The current study introduces grit as the outcome variable, offering a conceptually novel and complementary contribution to understanding how parental motivational climates relate to athletes’ psychological development. The present study sought to explore the associations between parent-initiated motivational climates and grit in male youth team sport players. The focus on grit as a dependent variable offers a novel contribution to the literature, as this construct has been scarcely examined in relation to parental motivational influences in sport. Accordingly, two main objectives were established: (1) to analyse the associative patterns of perceived mother and father motivational climates on the two dimensions of grit (perseverance of effort and consistency of interests) across different team sports, and (2) to examine whether these relationships differ according to sport type (basketball, handball, football, volleyball, and water polo). In light of AGT [22,25] and SDT [30,31], the following hypotheses were formulated: (1) A mastery-oriented motivational climate, characterised by an emphasis on learning, effort, and self-referenced improvement, will be positively associated with perseverance of effort and negatively associated with consistency of interests, as such environments have been associated with intrinsic and autonomous motivation [9,21]; (2) An ego-oriented or controlling motivational climate, focused on competition and external validation, will be negatively associated with perseverance of effort but positively associated with consistency of interests, as externally regulated motivation tends to be related to engagement only when socially reinforced [21]; (3) The strength and direction of these associations will vary across sports, consistent with contextual differences in parental involvement and the motivational structures of each sport [20,37].

## 2. Materials and Methods

### 2.1. Study Design

The present study drew upon data from the project “In Search of Excellence in Sport—a mixed-longitudinal study in young male athletes” (INEX). A comprehensive account of the INEX study protocol and design has been reported previously [48]. This project was designed to explore multiple psychological outcomes in youth athletes across various social domains. Thus, the present study constitutes a follow-up to a previously published investigation that examined the relationship between perceived parental motivational climates and fear of failure [43]. Both studies draw on the same dataset, specifically the measures of perceived mother- and father-initiated motivational climate, but pursue distinct research aims and theoretical frameworks. The current study introduces grit as a novel dependent variable, thereby offering a conceptually differentiated contribution to the literature on psychological development in sport. The decision to exclude grit-related data and analyses from the initial publication was made a priori, during the planning phase of the research project. This strategy aimed to ensure conceptual clarity and analytic focus in each manuscript. All grit-related data and analyses are original to the present study. We acknowledge the importance of full transparency in data reuse and affirm that this is the second and final planned publication based on this dataset. By conducting this follow-up study, we sought to generate complementary insights while maintaining scientific and methodological integrity.

### 2.2. Participants and Procedures

A total of 336 male youth athletes aged between 13 and 16 years (mean age = 14.14 ± 0.82 years) participated in this study. The sample included players from five team sports: basketball (n = 94), football (n = 119), handball (n = 35), volleyball (n = 58), and water polo (n = 30). Participants were recruited through purposive and convenience sampling criteria [49]. Players were considered “information-rich” [49] as they: (i) had a minimum of one year of sport-specific training and competitive involvement (typically more), (ii) were within the targeted adolescent developmental period, characterised by salient physical, cognitive, social, and emotional transitions relevant to identity formation and sport motivation, and (iii) were able and willing to engage in data collection. Recruitment occurred via clubs registered with their respective local sporting associations, with coaches and club coordinators identifying eligible athletes based on the defined criteria. The uneven distribution across sports reflects discipline-specific participation rates within the region and is therefore compatible with the purposive and convenience sampling approach employed. The resulting sample composition therefore mirrors the ecological reality of youth sport participation across the selected disciplines in the studied region.

Ethical approval for this study was obtained from the Research Ethics Committee of the first author’s affiliated institution, in accordance with the principles outlined in the Declaration of Helsinki (approval reference: CEFADE 13.2017). Both participants and their parents were fully informed about the study’s objectives, the extent of their involvement, and their right to withdraw at any stage without penalty. Confidentiality of the data was strictly maintained, and informed consent was obtained from all subjects and their legal guardians (parents).

### 2.3. Data Collection

Data collection was conducted in person, with participants completing a paper-based questionnaire in a quiet classroom environment under the supervision of a trained member of the research team. Questionnaires were administered in small groups of approximately 30 athletes, who responded individually. Prior to administration, participants were assured of confidentiality and anonymity, and received standardised verbal instructions. Clarifications regarding specific items or variables were provided when necessary. Completion of the questionnaire required approximately 30–40 min.

*Parent-Initiated Motivational Climate Questionnaire-2* (PIMCQ-2p). The Portuguese version of PIMCQ-2p [50] was employed to evaluate athletes’ perceptions of the motivational climate fostered by both their father and mother. This instrument comprises three subscales: one representing a mastery-oriented climate (learning/enjoyment climate; 8 items, e.g., “I feel that my mother/father is most satisfied when I learn something new in sport”) and two reflecting ego-oriented climates—the worry-conducive climate (5 items, e.g., “I feel that my mother/father makes me worried about losing in sport”) and the success-without-effort climate (5 items, e.g., “I feel that my mother/father is most satisfied when I succeed without effort in sport”). Responses are recorded on a 5-point Likert scale ranging from “strongly disagree” (1) and “strongly agree” (5). Santana and colleagues [50] reported the psychometric properties of the PIMCQ-2p, demonstrating the feasibility of the instrument. In the present sample, internal consistency was acceptable (Cronbach’s alpha = 0.875 to 0.885 for the mastery climate scale and Cronbach’s alpha = 0.756 to 0.815 for the ego climate scale).

*Short Grit Scale* (Grit-S). The Portuguese validated version of the Short-Grit Scale (Grit-S) was used [51] to assess Grit. The Grit-S includes 8 items grouped into two dimensions (4 items each): (a) consistency of interests (e.g., “I often set a goal but later choose to pursue a different one” [reverse score]), and (b) perseverance of effort (e.g., “I am a hard worker”). On a Likert scale ranging from 1 (not at all) to 5 (very much), participants rated the degree to which items corresponded to how they were typically. Coutinho and colleagues [51] reported the psychometric properties of the Grit-S, demonstrating the feasibility of the instrument. In the present sample, internal consistency was acceptable (Cronbach’s alpha = 0.726 and 0.744).

### 2.4. Data Analysis

Descriptive statistics were computed to obtain frequencies, percentages, means, and standard deviations. Assumptions of normality and homogeneity of variances were assessed using the Kolmogorov–Smirnov test and Levene’s test, respectively. Initially, Pearson’s product–moment correlation analyses were performed to explore the relationships between parent-initiated motivational climates (for both mother and father) and grit. Interpretation of the correlation coefficients followed Cohen’s [52] guidelines: values around r = 0.30 were considered small, r = 0.50 moderate, and r = 0.70 large. Subsequently, multiple linear regression analyses were carried out to investigate the extent to which parent-initiated motivational climates are associated with grit levels, both in general and across different sport disciplines. These analyses were first performed for the total sample and then repeated separately for each sport discipline (basketball, handball, football, volleyball, and water polo) to identify sport-specific associative patterns. The assumptions underlying multiple linear regression were evaluated and met, including linearity (through graphical inspection), independence of residuals (Durbin–Watson statistic), normality of residuals (Kolmogorov–Smirnov test), multicollinearity (assessed via Variance Inflation Factor and Tolerance values), and homoscedasticity (graphically examined). Regression assumption diagnostics (including Durbin–Watson, VIF, Tolerance, 95% confidence intervals, and Cook’s D) are summarised in Supplementary Table S1. All regression models were tested using an alpha level of 0.05, and exact *p*-values are reported. For *p*-values smaller than 0.001, results are presented as *p* < 0.001, in accordance with the recommendations of international statistical associations. Effect sizes were reported to contextualise the magnitude of the findings. For regression models, both the coefficient of determination (R^2^) and the adjusted R^2^ were reported, along with Cohen’s f^2^. According to Cohen’s [52] guidelines, f^2^ values of 0.02, 0.15, and 0.35 were interpreted as small, medium, and large effects, respectively. The analyses were carried out in SPSS, version 22.0, and statistical significance was set at *p* < 0.05 for all tests.

## 3. Results

To provide an overview of the study variables and to support the subsequent analyses, descriptive statistics and Pearson correlation coefficients were computed (Table 1). These analyses offered a preliminary examination of the associations between parental motivational climates and the two dimensions of grit, serving as the basis for testing the study hypotheses. A significant small and positive relationship between learning-enjoyment climate of mother (r = 0.271, *p* < 0.001) and father (r = 0.375, *p* < 0.001) and perseverance of effort was found. Concerning to mother success-without-effort climate, a significant small and negative relationship with the perseverance of effort (r = −0.169, *p* < 0.001) and a significant small and positive relationship with the consistency of interests (r = 0.270, *p* < 0.001) was found. In relation to the father-initiated motivational climate, a significant small and positive relationship between a worry-conducive climate (r = 0.268, *p* < 0.001) as well as a success-without-effort climate (r = 0.226, *p* < 0.001) and consistency of interests was found. Also, there was a significant small and negative relationship between father success-without-effort climate and perseverance of effort (r = −0.168, *p* < 0.001)

To address the first objective and test Hypotheses 1 and 2, multiple linear regression analyses were performed to examine the associations of perceived mother and father motivational climates and the two grit dimensions (perseverance of effort and consistency of interests) (Table 2). These analyses aimed to determine whether mastery- and ego-oriented climates are significantly associated with the athletes’ grit profiles. The total variance explained by the model when considering the influence of perceived mother and father-initiated motivational climate on the two dimensions of grit ranged between 8% and 16%. These values correspond to small to medium effect sizes (f^2^ ranging from 0.09 to 0.1), indicating that the models, although explaining a modest proportion of variance, represent meaningful relationships. Concerning the dimension of “Consistency of interests”, the perceived mother and father-initiated motivational climate had a similar variance explained (8%). This reflects a small effect size, suggesting a limited but significant association of parental motivational climate with this dimension of grit (f^2^ ranging approximately from 0.087 to 0.093). A mastery orientation (learning-enjoyment climate) of both mother and father was negatively associated with consistency of interests (β values of −0.085 and −0.041, respectively), while an ego orientation (worry-conducive and success-with effort climates) of both mother and father was positively associated with consistency of interests (β values ranging from 0.001 to 0.268). Regarding the dimension “Perseverance of effort”, the perceived climate created by father had a greater association with grit (16%) when compared to the perceived climate generated by mother (10%). These values correspond to medium and small effect sizes, respectively (f^2^ ≈ 0.19 for father, f^2^ ≈ 0.11 for mother), reinforcing that paternal motivational climate showed the most substantial statistical contribution in the overall model. The perceived learning-enjoyment and worry-conducive climates of both mother and father was positively associated with perseverance of effort (β values ranging from 0.012 and 0.370). A success-with-effort climate of both mother and father was negatively associated with this dimension of grit (β values of 0.159 to 0.163, respectively). This corresponds to small (f^2^ ≈ 0.11) and medium (f^2^ ≈ 0.19) effect sizes, respectively, as observed in the overall regression models.

To address the second objective and test Hypothesis 3, multiple linear regression analyses were conducted separately for each sport (basketball, football, handball, volleyball, and water polo) to examine the statistical associations of perceived maternal and paternal motivational climates on grit dimensions varied across disciplines (Table 3). This approach allowed us to explore sport-specific patterns in the associations between motivational climates and the two grit components. The perceived mother and father-initiated motivational climate is associated with the two dimensions of grit in basketball, football, and volleyball, but not in handball and water polo. The overall model accounted for 7 to 34% of the variance in basketball, 8 to 12% of the variance in football, and 12 to 18% of the variance in volleyball. A mastery orientation (learning-enjoyment climate) of both mother and father was positively associated with the “perseverance of effort” in basketball, football, and volleyball (β values ranging from 0.259 to 0.513) and “consistency of interests” in football and volleyball (β values ranging from 0.020 to 0.284). This dimension of parental motivational climate was negatively associated with “consistency of interests” in basketball (β values of −0.255 and −0.253, respectively). These R^2^ values correspond to small-to-large effect sizes (f^2^ ≈ 0.09–0.52), indicating that the predictive strength of parental motivational climates varied meaningfully across sports. Specifically, basketball showed large effects (f^2^ = 0.30–0.52), volleyball presented medium effects (f^2^ = 0.13–0.22), football displayed small-to-medium effects (f^2^ = 0.09–0.13), while handball and water polo revealed small or negligible effects (f^2^ = 0.11–0.20 and f^2^ = 0.11–0.30, respectively), despite some models not reaching statistical significance. This suggests that parental motivational climate associations were strongest in basketball, moderate in volleyball and football, and weak or nonsignificant in handball and water polo.

The perceived worry-conducive climate created by mother and father was positively associated with “perseverance of effort” in football (β values of 0.001 and 0.037, respectively) and negatively associated in basketball (β values of −0.035 and −0.150, respectively). In volleyball, the perceived father worry-conducive climate was positively associated with “perseverance of effort” (β = 0.271), while the perceived mother worry-conducive climate was negatively associated (β = −0.002). On the other hand, the worry-conducive climate created by mother and father was positively associated with “consistency of interests” in volleyball (β values of 0.109 and 0.200, respectively) and negatively associated in basketball (β values of −0.026 and −0.013, respectively). In football, the perceived father worry-conducive climate was positively associated with “consistency of interests” (β = 0.276), but the perceived mother worry-conducive climate was negatively associated with this dimension (β = −0.015). The perceived mother and father success-without-effort climate was negatively associated with “perseverance of effort” (β values ranging from −0.016 to −0.376) and positively associated with “consistency of interests” (β values ranging from 0.072 to 0.357) in basketball, football and volleyball. Overall, the magnitude of the effects indicates that the explanatory power of parental motivational climates was strongest in basketball (large effects), moderate in volleyball and football (medium effects), and weak in handball and water polo (small effects), supporting the interpretation that these associations may be context-dependent and sport-specific.

## 4. Discussion

The main purpose of the current study was to scrutinize the associations between the perceived mother- and father-initiated motivational climate and the level of grit exhibited by male youth participants engaged in team sports. In line with the first objective, this section initially discusses how distinct parental motivational orientations (mastery vs. ego) were differentially associated with the two grit dimensions (perseverance of effort and consistency of interests). Globally, the findings illustrated a significant association between the perceived mother- and father-initiated motivational climate and the manifestation of grit in the examined players. Moreover, the type of parental motivational climate orientation (i.e., mastery or ego orientation) was differently associated with grit predispositions. An ego-oriented climate (worry-conducive and success-with effort) initiated by both mothers and fathers was positively associated with consistency of interests, whereas a mastery-oriented climate (learning-enjoyment) created by them was negatively associated with this dimension of grit. Conversely, the perceived learning-enjoyment and worry-conducive climates initiated by both mothers and fathers were positively associated with perseverance of effort, while a success-with-effort climate created by them was negatively associated with this dimension of grit. Although the overall explained variance ranged from small to medium magnitudes, these values indicate practically meaningful relationships, suggesting that even modest parental influences may have some developmental relevance for athletes’ grit. These results partially align with previous findings demonstrating that mastery-oriented climates are generally associated with higher levels of persistence and intrinsic motivation [9,20,21], yet they also extend this knowledge by revealing that ego-involving climates may be tentatively linked with consistency of interests, as suggested by studies on competitive youth sport motivation [24,32,53]. This divergence highlights that the relationship between climate and grit may be more context-dependent than previously assumed.

Indeed, to date, no studies have differentiated the association between types of motivational climates and the various dimensions of grit. For example, previous studies have generally demonstrated that athletes endorsed in a mastery orientation (learning-approach goals) reported higher levels of grit [5,10,21]. However, this study not only revealed a positive association between ego orientation and grit but also identified a negative association between mastery orientation and one dimension of grit, specifically the consistency of interests. While the present findings suggest that ego-oriented climates may be associated with greater consistency of interests, this interpretation should be approached with caution. It is possible that the positive association reflects, at least in part, a perceived pressure to persist, driven by external expectations or fear of negative evaluation, rather than truly self-determined motivation. This perspective aligns with previous literature linking ego-involving climates to maladaptive outcomes such as anxiety, burnout, and perfectionism [29,46,54]. Therefore, the observed effect may be tentatively related to internalized performance pressure rather than genuine intrinsic consistency. Nonetheless, such climates may still be associated with sustained engagement, particularly when performance is socially valued and internalized by the athlete. Given that these associations presented small effect sizes, the findings should be interpreted as subtle but consistent trends rather than strong influencing factors of behaviour. Similar trends were noted by Appleton and colleagues [55,56] who found that perceived parental emphasis on winning was related to higher short-term persistence but lower long-term enjoyment, suggesting that persistence under ego pressure may not always correspond to adaptive grit.

On the other hand, a balance between a positive and enjoyable learning environment and a certain level of challenge or concern may be associated with children to persist in their efforts. The interplay of these factors underscores the importance of a balanced and supportive environment that encourages effort, acknowledges achievements, and possibly links to a positive learning experience for the development of grit in children. This interpretation aligns with Gao et al. [20], who highlighted that moderate parental autonomy support has been found to be associated with sustained engagement, whereas controlling climates have been linked with lower adaptive persistence. The medium effect sizes observed for perseverance of effort, particularly in relation to paternal climates, reinforce the practical relevance of these findings, as they are meaningful enough to be observable at the behavioural level. It is also important to consider possible confounding mechanisms that might explain why ego climates appear adaptive in this context [21,32]. Factors such as internalized competitiveness among young athletes, strong parental expectations regarding performance, or broader cultural norms that valorise achievement and persistence may be partly related to why ego-oriented climates appear beneficial. These elements could be tentatively linked with environments where children associate effort and success with personal validation and social approval, which may correspond with higher levels of grit indicators even in ego-driven contexts. Comparable patterns have been observed in educational settings, where externally regulated persistence often coexists with performance-oriented parental climates [57,58]. Future research should aim to disentangle these overlapping influences to better understand the nuanced association of motivational climates with psychological traits like grit. These findings complement previous work that examined how parental motivational climates relate to fear of failure [43], offering a broader perspective on how such climates may simultaneously influence both adaptive and maladaptive psychological dispositions in young athletes. This complementarity suggests that grit and fear of failure, although distinct in nature, may be tentatively linked with similar parental dynamics. Exploring the interplay between these constructs could help clarify how motivational climates may be linked with patterns associated with more or less adaptive development and offer a richer framework for understanding the dual impact of parenting practices in sport.

Of particular interest, and addressing the second objective, this study discloses a robust association between the perceived mother- and father-initiated motivational climate and the two dimensions of grit in different types of sport. Such associations were found for basketball, football, and volleyball, except for handball and water polo. This sport-dependent pattern is consistent with previous research showing that parental involvement and motivational influence tend to be more salient in sports with higher cultural visibility and social investment, such as football and basketball [37,59]. Several contextual and structural explanations may account for this pattern. Basketball, football, and volleyball are among the most culturally prominent and widely practised sports within the studied context, typically involving higher levels of parental engagement, public visibility, and competitive pressure during adolescence [37]. These sociocultural and organisational characteristics may amplify the salience of parental behaviours and expectations, which may be tentatively associated with the stronger salience of parental behaviours in relation to athletes’ motivational orientations and grit indicators. Conversely, handball and water polo, though sharing technical and tactical similarities as invasion games, often operate within smaller participation networks and receive comparatively lower media and social attention. The relative autonomy of athletes within these sports, coupled with potentially less direct parental oversight due to logistical or environmental factors (e.g., aquatic training settings), may be related to lower perceived motivational impact of parents. Thus, sport-specific ecological and cultural contexts may moderate the extent to which parental climates are associated with athletes’ psychological outcomes. The large effect sizes found in basketball further underline that, in highly competitive and socially salient sports, parental climates may be particularly strongly associated with players’ perseverance and consistency. Furthermore, the finding that parental mastery-oriented climates were negatively associated with consistency of interests in basketball echoes studies on early specialisation, suggesting that in highly performance-driven sports, learning-focused parental attitudes may conflict with athletes’ internalised competitive ethos [35,60]. Consequently, athletes perceiving their parents as encouraging exploration and enjoyment may exhibit lower sport-specific commitment but broader adaptability, a pattern that reflects flexible motivation rather than diminished grit.

Nevertheless, grit constitutes a complex psychological construct related to various situational factors [14,61]. Accordingly, the small-to-medium overall effect sizes observed indicate that while parental influence is meaningful, much of the variance in grit is associated with other contextual and intrapersonal variables. Consequently, while the motivational climate established by parents holds significance, it does not appear to be the sole correlate of grit dispositions in sports. This implies that additional influential factors may be associated with differences in grit among the mentioned players. For example, the study of Albert and colleagues [21] demonstrated that the motivational climate fostered by coaches was linked to a significant influence on how athletes perceive grit. This is in line with the findings of previous studies [24,59] showing that both parental and coach climates are jointly associated with perseverance and sport engagement among adolescents. This finding holds paramount significance as it has the potential to aid sports practitioners, such as sport psychologists, in recognizing the individual differences among athletes. It underscores the importance of offering tailored and precise guidance based on the unique sport context of each athlete. However, further investigations are warranted to delve into these matters within diverse sports contexts and cultures, aiming to offer a more comprehensive understanding of the intricacies surrounding parent-initiated motivation and its connections with grit.

It should also be acknowledged that although the proportion of explained variance across models ranged from approximately 7% to 34%, such values are consistent with those typically observed in psychological research, where complex behaviours are associated with multiple interacting determinants [4,11,17,21]. Despite the relatively modest explanatory power, the identification of statistical significance in these results has meaningful implications for applied practice. These findings suggest that parental influence constitutes an important, albeit partial, component of a broader developmental system that may be associated with the formation of grit. Accordingly, future studies should aim to incorporate additional predictors to enhance the explanatory power of the models and provide a more nuanced account of the factors influencing grit in youth athletes.

Despite the noteworthy findings presented, it is imperative to acknowledge the limitations inherent in this study. The research adopted a quantitative methodology, employing reliable and well-established instruments widely acknowledged in the literature. However, this approach primarily relies on the interpretation of participants’ self-reports and perceptions. Also, all variables were measured concurrently at a single time point; temporal precedence and causal direction cannot be determined. While these subjective insights are valuable, they benefit from triangulation with objective data to offer a comprehensive understanding of the associations perceived in parent-initiated motivational climates on grit predispositions. To address this, future research could incorporate qualitative approaches such as semi-structured interviews with parents to explore their self-perceived motivational strategies, or ethnographic observations during training sessions and competitions to examine real-time parental behaviors and interactions with athletes. Such methods would allow for a deeper examination of the social dynamics associated with the emergence of motivational climates and could help identify discrepancies between parents’ intentions and athletes’ perceptions. This integrated approach would contribute to a richer and more ecologically valid understanding of the developmental processes involved.

Furthermore, this study adopts a cross-sectional design, investigating the perceived parent-initiated motivational climate at a specific moment. Recognizing the potential temporal variability of motivational climates [27,32], future research should consider prospective longitudinal and/or experimental designs. Additionally, this study focuses on adolescent players, offering insights specific to this developmental phase. To comprehensively understand the relationship between parent-initiated motivational climates and grit, investigations should encompass athletes across various age periods, including childhood and adulthood. Moreover, extending the scope to different sports contexts, cultures, and female athletes is essential for a more expansive and nuanced understanding [4,14]. Although not the primary focus of this study, future research may benefit from considering athletes’ accumulated sport experience, as it could provide additional insight into how duration of participation may be associated with differences in perceived parental influence and psychological development. Moreover, the modest explanatory power observed (8–16%) may reflect, at least in part, the reduced number of participants in each sport-specific subsample, which may have constrained the statistical sensitivity to detect associations across disciplines. Also, it is noteworthy that this study solely reflects the perceptions of players regarding the perceived parent-initiated motivational climate and their own grit predispositions. To enrich this understanding, researchers should explore the perspectives of parents concerning the motivational climate they create and how these perceptions are associated with athletes’ grit indicators within the sporting context. Such investigations can shed light on the link between parental motivation, its behavioural association within the motivational climate, and the developmental origins of grit in their children.

Understanding the relationship between perceived parental and athletes’ grit offers valuable practical implications for coaches, sport psychologists, and those developing parental involvement strategies. Findings from this research may inform the design of more effective training frameworks, particularly through parent-focused educational initiatives aimed at encouraging adaptive motivational orientations, constructive communication, and supportive behaviors. Such interventions can be tailored to target specific long-term developmental outcomes [36], thereby being potentially associated with athletes’ grit and overall performance.

## 5. Conclusions

The present study contributes to a nuanced understanding of how parent-initiated motivational climates relate to young athletes’ grit across different team sports. The findings demonstrate that both mastery- and ego-oriented parental climates may be associated with perseverance and consistency of interests, although these associations appear context-dependent. Although the explained variance values and corresponding small-to-medium effect sizes indicate a modest association, they nonetheless reflect meaningful and practically relevant associations in the domain of youth sport, where multiple social and psychological factors interact. The evidence that certain sports (basketball, football, and volleyball) showed stronger associations than others (handball and water polo) highlights the importance of considering the cultural and structural features of each sport when examining the social ecology of motivation. In particular, the large effect sizes found for basketball underscore the practical significance of parental motivational climates in more competitive and socially salient sport contexts. These results collectively suggest that parental influence in sport is neither uniformly beneficial nor detrimental, but rather conditional upon how parental expectations, emotional tone, and involvement are perceived by athletes. Such findings extend the understanding of motivational climates by showing that grit may be associated with multiple pathways (some more adaptive than others) depending on the balance between autonomy support and performance pressure. While the study offers valuable insights, certain methodological aspects must be acknowledged. The sample comprised only male adolescent athletes, limiting generalisability and precluding gender-based analyses. Moreover, the cross-sectional design and moderate explanatory power advise caution in interpreting causal relationships. Future research should broaden the scope by incorporating female participants, adopting longitudinal or mixed-method approaches, and exploring how cultural and sport-specific factors may be associated with differences in the interplay between parental motivational climates and grit development. Ultimately, these findings encourage a critical reappraisal of parental involvement in youth sport, emphasising the need for climates that combine emotional support with realistic challenge, thereby being potentially associated with higher athletes’ perseverance without undermining their autonomy or intrinsic motivation.

## Figures and Tables

**Table 1:** Descriptive statistics, alpha coefficients, and Pearson product-moment correlations for perceived mother and father-initiated motivational climate and grit.

		Grit	
		Perseverance of Effort	Consistency of Interests
PIMC	Learning-enjoyment climate (mother)	0.271 ***	−0.092
	Worry-conducive climate (mother)	−0.017	0.016
	Success-without-effort climate (mother)	−0.169 ***	0.270 ***
	Learning-enjoyment climate (father)	0.375 ***	−0.088
	Worry-conducive climate (father)	−0.105	0.268 ***
	Success-without-effort climate (father)	−0.168 **	0.226 ***
	M ± Sd	3.93 ± 0.7	2.67 ± 0.9
	α	0.726	0.744

PIMC = Parent-Initiated Motivational Climate; *** *p* < 0.001 and ** *p* < 0.01 for statistical significance.

**Table 2:** Multiple linear regression analysis for perceived mother and father-initiated motivational climate and grit.

		Grit					
		Perseverance of Effort			Consistency of Interests		
		B	SE	β	B	SE	β
PIMC mother	Learning-enjoyment climate	0.274	0.53	0.268	−0.111	0.069	−0.085
	Worry-conducive climate	0.009	0.039	0.012	0.001	0.050	0.001
	Success-without-effort climate	−0.118	0.038	−0.163	0.249	0.049	0.268
R^2^		0.100			0.080		
Adjusted R^2^		0.092			0.072		
f^2^		0.111			0.087		
F		12.339			9.694		
*p*		<0.001 *			<0.001 *		
PIMC father	Learning-enjoyment climate	0.389	0.054	0.370	−0.055	0.072	−0.041
	Worry-conducive climate	0.031	0.043	0.043	0.187	0.058	0.198
	Success-without-effort climate	−0.109	0.039	−0.159	0.109	0.053	0.125
R^2^		0.161			0.085		
Adjusted R^2^		0.153			0.077		
f^2^		0.087			0.093		
F		21.412			10.334		
*p*		<0.001 *			<0.001 *		

PIMC = parent-initiated motivational climate; * *p* < 0.01 for statistical significance.

**Table 3:** Multiple linear regression analysis for perceived mother and father-initiated motivational climate and grit according to the type of sport.

	Parent-Initiated Motivational Climate		Grit					
			Perseverance of Effort			Consistency of Interests		
			B	SE	β	B	SE	β
Basketball	Mother	Learning-enjoyment climate	0.529	0.113	0.436	−0.354	0.140	−0.255
		Worry-conducive climate	−0.036	0.094	−0.035	−0.030	0.117	−0.026
		Success-without-effort climate	−0.131	0.080	−0.152	0.108	0.099	0.110
	R^2^		0.229			0.087		
	Adjusted R^2^		0.203			0.057		
	f^2^		0.297			0.095		
	F		9.113			2.927		
	*p*		<0.001 **			0.038 *		
	Father	Learning-enjoyment climate	0.493	0.083	0.513	−0.277	0.113	−0.253
		Worry-conducive climate	−0.144	0.085	−0.150	−0.014	0.116	−0.013
		Success-without-effort climate	−0.075	0.073	−0.089	0.068	0.099	0.072
	R^2^		0.342			0.071		
	Adjusted R^2^		0.320			0.040		
	f^2^		0.520			0.076		
	F		15.912			2.930		
	*p*		<0.001 **			0.041 *		
Handball	Mother	Learning-enjoyment climate	−0.085	0.234	−0.065	−0.517	0.265	−0.348
		Worry-conducive climate	−0.070	0.174	−0.073	−0.063	0.197	−0.058
		Success-without-effort climate	−0.373	0.158	−0.416	0.211	0.179	0.208
	R^2^		0.168			0.162		
	Adjusted R^2^		0.087			0.081		
	f^2^		0.202			0.193		
	F		1.889			1.806		
	*p*		0.154			0.169		
	Father	Learning-enjoyment climate	0.197	0.240	0.151	−0.053	0.292	−0.036
		Worry-conducive climate	0.090	0.189	0.104	0.394	0.230	0.405
		Success-without-effort climate	−0.462	0.159	−0.572	−0.017	0.193	−0.019
	R^2^		0.289			0.170		
	Adjusted R^2^		0.220			0.090		
	f^2^		0.406			0.205		
	F		3.797			1.908		
	*p*		0.061			0.151		
Football	Mother	Learning-enjoyment climate	0.265	0.085	0.278	0.089	0.116	0.068
		Worry-conducive climate	0.001	0.061	0.001	−0.014	0.083	−0.015
		Success-without-effort climate	−0.039	0.063	−0.055	0.304	0.085	0.314
	R^2^		0.083			0.098		
	Adjusted R^2^		0.059			0.074		
	f^2^		0.091			0.109		
	F		3.584			4.287		
	*p*		0.016 *			0.007 *		
	Father	Learning-enjoyment climate	0.386	0.101	0.346	0.068	0.140	0.045
		Worry-conducive climate	0.025	0.067	0.037	0.250	0.093	0.276
		Success-without-effort climate	−0.011	0.069	−0.016	0.070	0.095	0.075
	R^2^		0.117			0.096		
	Adjusted R^2^		0.094			0.072		
	f^2^		0.133			0.106		
	F		5.199			4.186		
	*p*		0.002 *			0.007 *		
Volleyball	Mother	Learning-enjoyment climate	0.205	0.106	0.259	0.022	0.144	0.020
		Worry-conducive climate	−0.001	0.082	−0.002	0.094	0.111	0.109
		Success-without-effort climate	−0.169	0.083	−0.270	0.309	0.112	0.357
	R^2^		0.115			0.146		
	Adjusted R^2^		0.066			0.099		
	f^2^		0.130			0.171		
	F		2.903			3.011		
	*p*		0.041 *			0.038 *		
	Father	Learning-enjoyment climate	0.319	0.123	0.326	0.383	0.170	0.284
		Worry-conducive climate	0.170	0.097	0.271	0.173	0.134	0.200
		Success-without-effort climate	−0.208	0.086	−0.376	0.105	0.119	0.137
	R^2^		0.173			0.178		
	Adjusted R^2^		0.127			0.132		
	f^2^		0.209			0.217		
	F		3.702			3.836		
	*p*		0.017 *			0.015 *		
Water Polo	Mother	Learning-enjoyment climate	0.446	0.235	0.345	−0.055	0.327	−0.031
		Worry-conducive climate	−0.001	0.105	−0.001	−0.106	0.146	−0.135
		Success-without-effort climate	0.088	0.110	0.146	0.219	0.153	0.265
	R^2^		0.126			0.103		
	Adjusted R^2^		0.025			−0.001		
	f^2^		0.144			0.115		
	F		1.298			1.033		
	*p*		0.295			0.394		
	Father	Learning-enjoyment climate	0.381	0.191	0.360	−0.275	0.247	−0.190
		Worry-conducive climate	−0.092	0.168	−0.139	0.077	0.217	0.085
		Success-without-effort climate	0.114	0.145	0.202	0.278	0.188	0.357
	R^2^		0.136			0.231		
	Adjusted R^2^		0.036			0.142		
	f^2^		0.157			0.300		
	F		1.422			2.711		
	*p*		0.258			0.065		

** *p* < 0.01 and * *p* < 0.05 for statistical significance.

## Data Availability

The data presented in this study are available on request from the corresponding author due to the confidentiality and anonymity of participants’ responses.

## Supplementary Materials

The following supporting information can be downloaded at: https://www.mdpi.com/article/10.3390/sports13120421/s1, Table S1: Regression diagnostics for all models testing the predictive effects of perceived mother- and father-initiated motivational climates on grit dimensions.

## References

[1] Duckworth A.L., Peterson C., Matthews M.D., Kelly D.R. (2007). Grit: Perseverance and Passion for Long-Term Goals. J. Personal. Soc. Psychol..

[2] Duckworth A.L., Quinn P.D. (2009). Development and validation of the short grit scale (grit–S). J. Personal. Assess..

[3] Duckworth A.L., Gross J.J. (2014). Self-control and grit related but separable determinants of success. Curr. Dir. Psychol. Sci..

[4] Cormier D.L., Fergusson L.J., Gyurcsik N.C., Briere J.L., Dunn J.G., Kowalski K.C. (2021). Grit in sport: A scoping review. Int. Rev. Sport Exerc. Psychol..

[5] Shen Y., Sim Y., Bae J., Bang J., Seo J. (2025). Differences in the relationship among grit, self-regulation, competition preparation, and sport confidence across performance levels: A multi-group analysis. Sci. Rep..

[6] Barcza-Renner K., Shipher A., Basevitch I. (2025). An examination of the relationship between burnout and grit in college athletes. Asian J. Sport Exerc. Psychol..

[7] Ionel M.S., Ion A., Visu-Petra L. (2023). Personality, grit, and performance in rockclimbing: Down to the nitty-gritty. Int. J. Sport Exerc. Psychol..

[8] Larkin P., O’Connor D., Williams A.M. (2016). Does grit influence sport-specific engagement and perceptual-cognitive expertise in elite youth soccer?. J. Appl. Sport Psychol..

[9] Albert E., Petrie T.A., Moore W.G. (2021). The relationship of motivational climates, mindsets, and goal orientations to grit in male adolescent soccer players. Int. J. Sport Exerc. Psychol..

[10] Kovács K.E. (2025). Athlete profiles along grit, sport orientation and sport persistence based on a quantitative research on Hungarian athletes. Front. Sports Act. Living.

[11] Cormier D.L., Fergusson L.J., Gyurcsik N.C., Briere J.L., Mosewich A.D., Kowalski K.C. (2024). A quantitative assessment of the predictive utility of grit in sport. Psychol. Sport Exerc..

[12] Cocić D., Larkin P., Hendry D.T., Williams A.M., O’Connor D., Bilalić M. (2024). How small differences grow over time—The snowball effect of grit on practice in sport. Int. J. Sport Exerc. Psychol..

[13] Larkin P., Cocic D., Hendry D.T., Williams A.M., O’Connor D., Bilalic M. (2023). Gritting One’s way to success—Grit explains skill in elite youth soccer players beyond (deliberate) practice. Psychol. Sport Exerc..

[14] Nothnagle E.A., Knoester C. (2025). Sport participation and the development of Grit. Leis. Ciences.

[15] Tedesqui R.A., Young B.W. (2018). Comparing the contribution of conscientiousness, self-control, and grit to key criteria of sport expertise development. Psychol. Sport Exerc..

[16] Tedesqui R.A., Young B.W. (2017). Investigating grit variables and their relations with practice and skill groups in developing sport experts. High Abil. Stud..

[17] Apró A., Fejes N., Bandi S.A., Járai R. (2024). Investigating the effect of grit trait on performance and success in Hungarian athlete’s sample. Front. Psychol..

[18] Gray H.M., Moran R.N., Elder E., Wilkerson A., Chaney E., Gilmore-Childress G., Wallace J. (2023). Grit, athlete burnout, and well-being in female collegiate student-athletes. J. Athl. Train..

[19] Robertson-Kraft C., Duckworth A.L. (2014). True grit: Trait-level perseverance and passion for long-term goals predicts effectiveness and retention among novice teachers. Teach. Coll. Rec..

[20] Gao Z., Chee C.S., Wazir M., Wang J., Zheng X., Wag T. (2025). The role of parents in the motivation of young athletes: A systematic review. Front. Psychol..

[21] Albert E., Petrie T.A., Moore W.G. (2022). Achievement motivation and grit’s perseverance of effort among collegiate athletes. Int. J. Sport Exerc. Psychol..

[22] Nicholls J.G. (1989). Competence and Accomplishment: A Psychology of Achievement Motivation.

[23] Smith R.E., Smoll F.L., Cumming S.P. (2009). Motivational climate and changes in young athletes’ achievement goal orientations. Motiv. Emot..

[24] Atkins M.R., Johnson D.M., Force E.C., Petrie T.A. (2015). Peers, parents, and coaches, oh my! The relation of the motivational climate to boys’ intention to continue in sport. Psychol. Sport Exerc..

[25] Ames C., Roberts G. (1995). Achievement goals, motivational climate, and motivational processes. Motivation in Sport and Exercise.

[26] Roberts G.C., Roberts G.C., Treasure D.C. (2012). Motivation in sport and exercise from an achievement goal theory perspective: After 30 years, where are we?. Advances in Motivation in Sport and Exercise.

[27] Vitali F., Bortoli L., Bertinato L., Robazza C., Schena F. (2015). Motivational climate, resilience, and burnout in youth sport. Sport Sci. Health.

[28] Leo F.M., Sánchez-Miguel P.A., Sánchez-Oliva D., Amado D., García-Calvo T. (2015). Motivational climate created by other significant actors and antisocial behaviors in youth sport. Kinesiology.

[29] Gustafsson H., Hill A.P., Stenling A., Wagnsson S. (2016). Profiles of perfectionism, parental climate, and burnout among competitive junior athletes. Scand. J. Med. Sci. Sports.

[30] Deci E.L., Ryan R.M. (1985). Intrinsic Motivation and Self-Determination in Human Behavior.

[31] Deci E.L., Ryan R.M. (2000). The “what” and “why” of goal pursuits: Human needs and the self-determination of behavior. Psychol. Inq..

[32] Harwood C., Keegan R., Smith J., Raine A. (2015). A systematic review of the intrapersonal correlates of motivational climate perceptions in sport and physical activity. Psychol. Sport Exerc..

[33] Chan D.K., Lonsdale C., Fung H. (2012). Influences of coaches, parents, and peers on the motivational patterns of child and adolescent athletes. Scand. J. Med. Sci. Sports.

[34] Akin A., Arslan S. (2014). The relationships between achievement goal orientations and grit. Egit. Ve Bilim.

[35] Harwood C., Knight C., Thrower S., Berrow S.R. (2019). Advancing the study of parental involvement to optimise the psychosocial development and experiences of young athletes. Psychol. Sport Exerc..

[36] Knight C., Berrow S.R., Harwood C. (2017). Parenting in sport. Curr. Opin. Psychol..

[37] Dorsch T.E., Wright E., Eckardt V.C., Elliot S., Thrower S., Knight C. (2021). A history of parent involvement in organized youth sport: A scoping review. Sport Exerc. Perform. Psychol..

[38] Harwood C., Knight C. (2015). Parenting in youth sport: A position paper on parenting expertise. Psychol. Sport Exerc..

[39] Barber H., Sukhi H., White S. (1999). The influence of parent-coaches on participant motivation and competitive anxiety in youth sport participants. J. Sport Behav..

[40] Lemyre P., Treasure D.C., Roberts G.C. (2006). Influence of variability in motivation and affect on elite athlete burnout susceptibility. J. Sport Exerc. Psychol..

[41] Côté J. (1999). The influence of the family in the development of talent in sport. Sport Psychol..

[42] Coutinho P., Mesquita I., Fonseca A.M. (2016). Talent development in sport: A critical review of pathways to expert performance. Int. J. Sports Sci. Coach..

[43] Coutinho P., Bessa C., Dias C., Mesquita I., Fonseca A.M. (2024). Unveiling players’ perceptions of mother- and father-initiated motivational climates and fear of failure in youth male team sports. Sports.

[44] MacDonald D., Côté J., Eys M., Deakin J. (2011). The Role of Enjoyment and Motivational Climate in Relation to the Personal Development of Team Sport Athletes. Sport Psychol..

[45] Dorsch T., Smith A.L., Dotterer A.M. (2016). Individual, relationship, and context factors associated with parent support and pressure in organized youth sport. Psychol. Sport Exerc..

[46] O’Rourke D., Smith R.E., Smoll F.L., Cumming S.P. (2014). Relations of parent- and coach-initiated motivational climates to young athletes’ self-esteem, performance anxiety, and autonomos motivation: Who is more influential?. J. Appl. Sport Psychol..

[47] Rhodes R.E., Stearns J., Berry T., Faulkner G., Latimer-Cheung A.E., O’Reilly N., Tremblay M.S., Vanderloo L., Spence J.C. (2019). Predicting parental support and parental perceptions of child and youth movement behaviors. Psychol. Sport Exerc..

[48] Guimarães E., Baxter-Jones A., Williams A.M., Tavares F., Janeira M.A., Maia J. (2021). The role of growth, maturation and sporting environment on the development of performance and technical and tactical skills in youth basketball players: The INEX study. J. Sports Sci..

[49] Patton M.Q. (2015). Qualitative Research and Evaluation Methods.

[50] Santana P., Figueiras T., Dias C., Corte-Real N., Brustad R., Fonseca A.M. (2010). Versão portuguesa do Parent-Initiated Motivation Questionnaire—2 (PIMCQ-2p). Rev. Port. De Ciências Do Desporto.

[51] Coutinho P., Bessa C., Ramos A., Dias C., Mesquita I., Fonseca A.M. (2022). Validação da Short Grit Scale através de uma Análise Fatorial Confirmatória para uma população de jovens atletas portugueses do sexo masculino. Rev. Port. De Ciências Do Desporto.

[52] Cohen J. (1988). Statistical Power Analysis for Behavioral Sciences.

[53] Wagnsson S., Stenling A., Gustafsson H., Augustsson C. (2016). Swedish youth football players’ attitudes towards moral decision in sport as predicted by the parent-initiated motivational climate. Psychol. Sport Exerc..

[54] O’Rourke D., Smith R.E., Smoll F., Cumming S.P. (2011). Trait anxiety in young athletes as a function of parental pressure and motivational climate: Is parental pressure always harmful?. J. Appl. Sport Psychol..

[55] Appleton P.R., Hall H.K., Hill A.P. (2010). Family patterns of perfectionism: An examination of elite junior athletes and their parents. Psychol. Sport Exerc..

[56] Appleton P.R., Hall H.K., Hill A.P. (2011). Examining the influence of the parent-initiated and coach-created motivational climates upon athletes’ perfectionistic cognitions. J. Sports Sci..

[57] Cox A.E., Ullrich-French S. (2010). The motivational relevance of peer and teacher relationship profiles in physical education. Psychol. Sport Exerc..

[58] Hagger M., Chatzisarantis N., Hein V., Soós I., Karsai I., Lintunen T., Leemans S. (2009). Teacher, peer and parent autonomy support in physical education and leisure-time physical activity: A transcontextual model of motivation in four nations. Psychol. Health.

[59] Keegan R., Spray C., Harwood C., Lavallee D. (2010). The motivational atmosphere in youth sport: Coach, parent, and peer influences on motivation in specializing sport participants. J. Appl. Sport Psychol..

[60] Coutinho P., Ribeiro J., Mesquita da Silva S., Fonseca A.M., Mesquita I. (2021). The influence of parents, coaches, and peers in the long-term development of highly skilled and less skilled volleyball players. Front. Psychol..

[61] Cormier D.L., Jorgensen H., Ferguson L.J., Gyurcsik N.C., Briere J.L., Kowalski K.C. (2025). Constructing a grounded theory of grit in sport: Understanding the development and outcomes of long-term passion and perseverance in competitive athletes. Qual. Res. Sport Exerc. Health.

