# Supplementary material for: Parental Involvement in Sport: Mother- and Father-Initiated Motivational Climates and Their Associations with Grit in Youth Male Team Sport Players
Source: Sports (Basel). 2025 Dec 1;13(12):421. doi: 10.3390/sports13120421 (PMC12736585; doi:10.3390/sports13120421)
Supplement: Supplementary file 1 [file sports-13-00421-s001.zip › sports-3958156-supplementary.pdf]

**Supplementary Table S1. Regression diagnostics for all models testing the predictive effects of perceived mother- and father-initiated motivational climates on grit dimensions.**

| Model | Durbin–Watson | VIF (Range) | Tolerance (Range) | 95% CI for β (Range) | Cook's D (Mean / Max) | Assumption Verification Summary |
|---|---|---|---|---|---|---|
| Overall Model (All sports combined) | 1.98 | 1.02–2.21 | 0.45–0.98 | −0.22 to 0.41 | 0.02 / 0.10 | All assumptions met; residuals randomly distributed, no multicollinearity, independence verified. |
| Basketball | 1.89 | 1.05–2.18 | 0.46–0.95 | −0.26 to 0.53 | 0.03 / 0.12 | No violation of normality or homoscedasticity; no influential cases detected. |
| Football | 2.07 | 1.01–2.03 | 0.49–0.99 | −0.21 to 0.39 | 0.02 / 0.09 | Residual plots indicate linearity and homoscedasticity; Cook's D below 0.15 for all cases. |
| Handball | 1.91 | 1.08–2.25 | 0.44–0.93 | −0.37 to 0.46 | 0.03 / 0.11 | Variance inflation within acceptable range; no autocorrelation detected. |
| Volleyball | 2.05 | 1.02–2.10 | 0.48–0.98 | −0.27 to 0.51 | 0.02 / 0.08 | Assumptions of independence and normality met; residuals evenly distributed. |
| Water Polo | 1.94 | 1.03–2.30 | 0.43–0.97 | −0.35 to 0.47 | 0.03 / 0.13 | Slight heteroscedasticity observed, but within acceptable tolerance; no influential points. |

Note: Durbin–Watson statistics between 1.5 and 2.5 indicate independence of residuals. All VIF values were below 5.0 (and most below 2.5), confirming the absence of problematic multicollinearity. Tolerance values above 0.40 further support this conclusion. Cook's distance values remained well below the conservative threshold of 1.0, indicating no

influential cases. Residual and P–P plots visually confirmed normality and homoscedasticity across all models.
